# Last Bacteria Standing: VREfm Persistence in the Hospitalized Gut

**DOI:** 10.1128/mbio.00670-22

**Published:** 2022-06-28

**Authors:** Madison E. Stellfox, Daria Van Tyne

**Affiliations:** a Division of Infectious Diseases, University of Pittsburgh School of Medicine, Pittsburgh, Pennsylvania, USA

**Keywords:** Enterococcus, antibiotic resistance, whole genome sequencing

## Abstract

Enterococci are gram-positive, gastrointestinal (GI) tract commensal bacteria that have recently evolved into multidrug-resistant nosocomial pathogens. Enterococci are intrinsically hardy, meaning that they can thrive in challenging environments and outlast other commensal bacteria. Further adaptations enable enterococci to dominate the GI tracts of hospitalized patients, and this domination precedes invasive infection and facilitates transmission to other patients. A recent study by Boumasmoud et al. used whole genome sequencing (WGS) to characterize 69 vancomycin-resistant Enterococcus faecium (VREfm) isolates collected from a Swiss hospital. WGS uncovered a clone that was repeatedly sampled from dozens of patients over multiple years. This persistent clone accumulated mutations as well as a novel linear plasmid, which together likely increased its persistence in the GI tracts of infected patients. This study is one of several recent examples that highlight the genetic plasticity of VREfm as it adapts to the hospitalized gut and becomes a leading nosocomial pathogen.

## COMMENTARY

**FIG 1:**
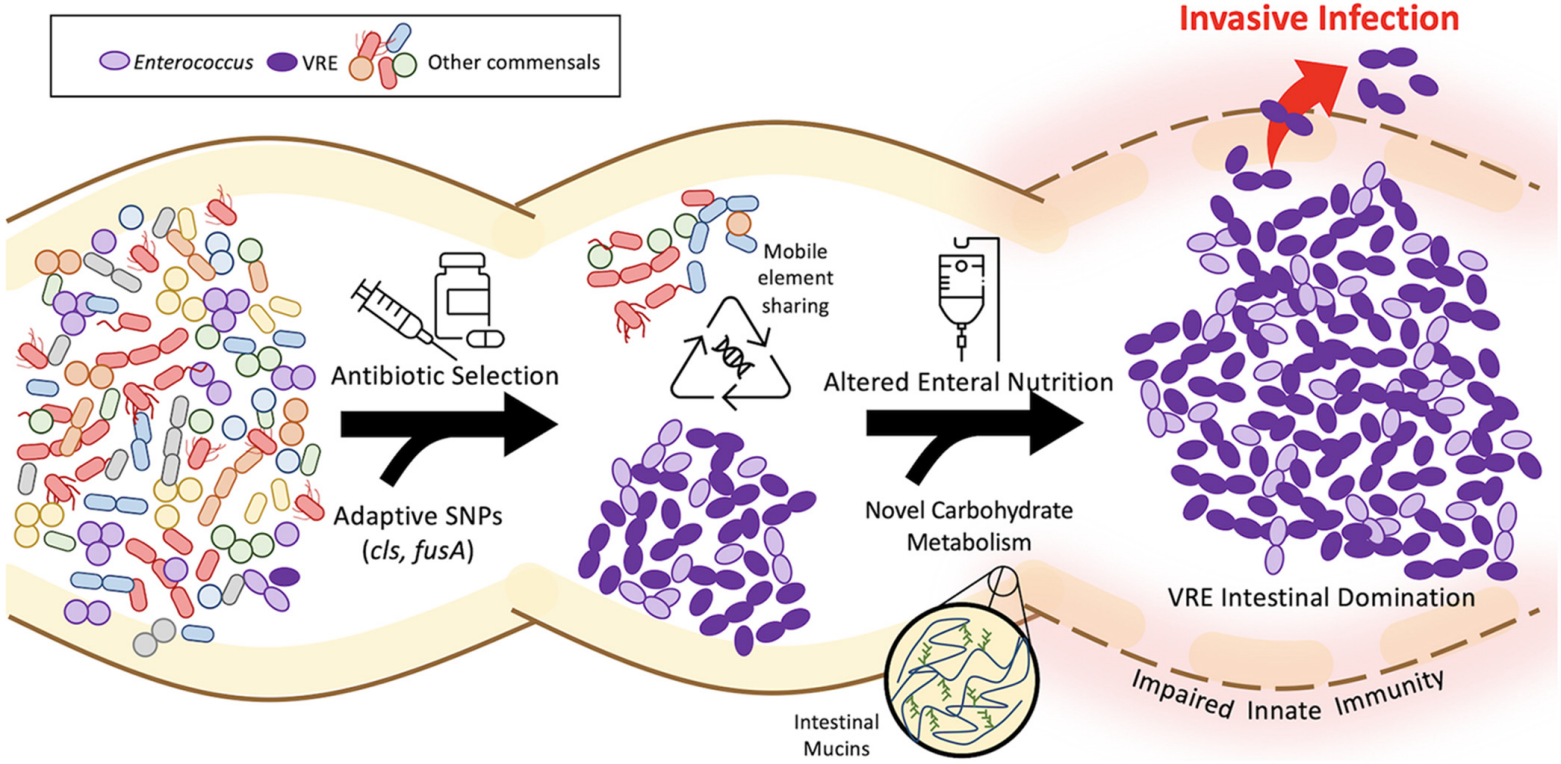
Model showing how VRE genetic plasticity may lead to intestinal domination and invasive infection. Brown curved lines indicate the mucosal surface of the gastrointestinal (GI) tract, and light-yellow shading depicts the associated mucus layer. The right-most portion highlights inflammatory changes that can occur at the GI mucosal surface in times of illness, which can lead to a breakdown of innate host defenses. Black horizontal arrows are labeled with examples of external selective pressures, such as antibiotic exposure and altered enteral nutrition. Curved arrows highlight genetic adaptations that can occur during selection to generate a population of enterococci (purple ovals) that resists eradication and outcompetes other enteric commensal bacteria (depicted by multicolored shapes).

Enterococci are GI tract commensals of nearly all terrestrial animals, from insects to humans (1, 2). In the antibiotic era, enterococci have emerged as prominent pathogens that cause antibiotic-resistant infections in vulnerable hosts. A prime example is vancomycin-resistant Enterococcus faecium (VREfm), a hospital-associated pathogen that readily colonizes the antibiotic-perturbed GI tract and also causes deadly systemic infections. VREfm colonization and infection are facilitated by the fact that enterococci are often the “last bacteria standing” after other members of the GI flora are removed via broad-spectrum antibiotics and other treatments that deplete commensal flora, such as alterations in enteral nutrition, gastric acid suppression, skin and oropharyngeal decontamination, as well as mucosal and skin barrier disruption (3). The ability of VREfm to persist in the GI tract stems from the fact that enterococci are intrinsically hardy, meaning that they can tolerate a variety of cellular stresses that other bacteria cannot (1, 4). This persistence enables further adaptation through mutations and acquisition of mobile elements, both of which allow VREfm to increase their abundance in the GI tract and ultimately cause infection (Fig. 1) (5).

Whole genome sequencing (WGS) is a powerful tool for studying bacterial adaptation and identifying outbreaks of drug-resistant pathogens. The study by Boumasmoud et al. used WGS to sample VREfm at a single Swiss hospital over a 4-year period (6). Sequencing of 69 VREfm colonizing and invasive isolates collected from 61 different patients uncovered a persistent clone belonging to multilocus sequence type (ST) 203 that was sampled 31 times over 3 years and was responsible for an outbreak of VREfm infection. The authors looked for single nucleotide polymorphisms (SNPs) that were shared among all outbreak isolates but not other sampled ST203 isolates, and identified 6 nonsynonymous SNPs that were unique to the outbreak isolates. Among the six mutated genes, at least two resided in pathways that have previously been associated with bacterial survival in the face of VREfm-directed antimicrobial therapy. The first mutated gene is the cardiolipin synthase (*cls*), which was found to encode an Ala20Asp mutation among the outbreak isolates. Mutations in *cls* are known to arise in *Enterococcus* genomes following treatment with daptomycin, one of the few available antibiotic options to treat vancomycin-resistant enterococcal infections. Daptomycin disrupts the integrity of the bacterial cell membrane, and mutations in *cls* alter phospholipid biosynthesis and decrease bacterial susceptibility to this agent (7). Isolates in the outbreak clade harboring the Ala20Asp mutation in *cls* were found to have increased daptomycin MICs compared to the ancestral strain. Notably, two prior studies identified the same mutation in *cls* as likely selected following daptomycin exposure (8, 9). While this *cls* mutation alone does not appear to confer overt resistance to daptomycin, one of these earlier studies showed that *cls* mutations were associated with increased daptomycin tolerance, as measured by minimum bactericidal concentration (MBC) testing (9). Antibiotic tolerance is increasingly appreciated as a clinically relevant phenotype that contributes to persistent and recurrent infections (10). Convergent evolution of the same *cls* mutation in VREfm across three separate studies strongly suggests that this particular mutation is important for bacterial tolerance during daptomycin selection, regardless of whether the mutation confers true resistance or not.

Antibiotic tolerance can also be achieved through induction of the stringent response, a signaling cascade that globally alters transcriptional and translational profiles to downregulate metabolism and slow bacterial growth (11). Mutations in the (p)ppGpp synthetase, *Rel*, as well as other effector proteins, are associated with antibiotic tolerance and persistent bacterial infections (12). In the study by Boumasmoud et al., all outbreak isolates encoded a Val678Ile mutation in FusA, which is also known as elongation factor G (EF-G). EF-G catalyzes the GTP-dependent ribosomal translocation step during translation and is a binding partner of (p)ppGpp, which helps propagate the decreased translational activity when the stringent response is activated (13). While it is uncertain what the effects of this particular mutation in FusA might be, the authors observed that the growth rates of outbreak isolates were significantly slower than those of ancestral strains. A slow growth rate is one of the hallmark phenotypes of an activated stringent response, and it is believed that slow growth helps bacterial populations survive stress by slowing down metabolism. These results cause us to wonder whether the outbreak clade-defining chromosomal mutation in FusA could induce or modulate the stringent response and contribute to an antibiotic tolerance phenotype. Mutations that cause activation of the stringent response have been previously observed to evolve during persistent infection, including in VREfm (14, 15). Genetic signatures of stringent response activation can be difficult to detect because there is no single gene or causal mutation. Nonetheless, it is becoming increasingly clear that activation of this pathway is a conserved mechanism enabling bacterial persistence in harsh environments, such as the hospital environment or the GI tracts of hospitalized patients. This persistence offers additional time and opportunities for bacteria to adapt through mutation and mobile element acquisition.

The GI tract of the hospitalized patient differs significantly from the healthy human gut and provides a vastly different metabolic environment for intestinal flora. Prior genomic analyses of hospital-adapted enterococci have suggested that these pathogens have acquired genes that allow them to access and metabolize nutrients that commensal enterococci cannot. One prominent example of this is the enrichment of genes encoding phosphotransferase systems (PTSs) and other carbohydrate utilization enzymes among hospital-adapted E. faecium (16, 17). In another study, a unique PTS that allows for the utilization of amino sugars, which occur on epithelial cell surfaces and mucin, was shown to enhance the ability of E. faecium to colonize the antibiotic-perturbed GI tract (18). In the study by Boumasmoud et al., the outbreak isolates acquired the ability to metabolize N-acetyl-galactosamine (GalNac), a component of intestinal mucin, due to an operon encoded on a novel linear plasmid. As noted by the authors, this plasmid was similar to other recently described linear E. faecium plasmids (19, 20). While the linear plasmid observed in this study was similar in genetic and topological structure to those previously reported, the GalNac metabolism operon observed here was not present in the other E. faecium linear plasmids. The authors showed that the linear plasmid carrying the GalNAc metabolism operon gave VREfm the ability to grow in the presence of GalNAc, and suggested that this plasmid might have enabled VREfm to more efficiently colonize the GI tracts of hospitalized patients.

Where might this GalNAc operon have originated? To investigate this question, we searched for the ~12.5 kb GalNAc operon sequence in the NCBI database using nucleotide BLAST and identified significant homology spanning the entire length of the operon to two Enterococcus avium genomes. The two genomes were *E. avium* strain 352 (GenBank accession CP034169.1), which was isolated from a patient with cholelithiasis in China in 2018, and *E. avium* strain FDAARGOS_184 (GenBank accession CP024590.1), which was isolated from an abscess in a pediatric patient in the United States in 2015. The operon in both *E. avium* genomes resided on the chromosome and lacked identifiable mobile element signatures in the flanking regions. Nonetheless, the sequence of this GalNAc operon in the outbreak linear plasmid and the two *E. avium* genomes shared over 99% nucleotide identity, strongly suggesting the possible origin of this operon in the *E. avium* genome. Prior studies by Hashimoto et al. showed that linear E. faecium plasmids could be readily transferred between different enterococcal species, including E. faecium, E. faecalis, *E. casseliflavus*, and *E. hirae* (19, 20). Taken together, these observations suggest that the GalNAc operon observed in this study might have originated in *E. avium*, been transferred to a mobile element, such as a linear plasmid, and then moved into E. faecium, where it was observed by the study authors.

Overall, the study by Boumasmoud et al. highlights the benefits of using WGS in conjunction with hospital epidemiology to detect bacterial clones causing outbreaks among hospitalized patients. Additionally, WGS is a useful tool for identifying ways that enterococci adapt to the antibiotic-perturbed GI tract. In this case, adaptations that enhanced antibiotic tolerance, contributed to persistent infection, and conferred the ability to metabolize new food sources likely caused the success of this particular clone. While enterococci are intrinsically hardy, this study shows how VREfm continually adapt to persist in the hospitalized GI tract, where they can remain poised to cause systemic disease.
